# BRCA1/2 genetic background-based therapeutic tailoring of human ovarian cancer: hope or reality?

**DOI:** 10.1186/1757-2215-2-14

**Published:** 2009-10-13

**Authors:** Pierosandro Tagliaferri, Monica Ventura, Francesco Baudi, Iole Cucinotto, Mariamena Arbitrio, Maria Teresa Di Martino, Pierfrancesco Tassone

**Affiliations:** 1 Medical Oncology Unit and Center for Genetic Counseling and Innovative Treatments, Tommaso Campanella Cancer Center,Catanzaro 88100, Italy; 2 Magna Græcia University Campus Salvatore Venuta, Catanzaro 88100, Italy

## Abstract

Ovarian epithelial tumors are an hallmark of hereditary cancer syndromes which are related to the germ-line inheritance of cancer predisposing mutations in BRCA1 and BRCA2 genes. Although these genes have been associated with multiple different physiologic functions, they share an important role in DNA repair mechanisms and therefore in the whole genomic integrity control. These findings have risen a variety of issues in terms of treatment and prevention of breast and ovarian tumors arising in this context. Enhanced sensitivity to platinum-based anticancer drugs has been related to BRCA1/2 functional loss. Retrospective studies disclosed differential chemosensitivity profiles of BRCA1/2-related as compared to "sporadic" ovarian cancer and led to the identification of a "BRCA-ness" phenotype of ovarian cancer, which includes inherited BRCA1/2 germ-line mutations, a serous high grade histology highly sensitive to platinum derivatives. Molecularly-based tailored treatments of human tumors are an emerging issue in the "era" of molecular targeted drugs and molecular profiling technologies. We will critically discuss if the genetic background of ovarian cancer can indeed represent a determinant issue for decision making in the treatment selection and how the provocative preclinical findings might be translated in the therapeutic scenario. The presently available preclinical and clinical evidence clearly indicates that genetic background has an emerging role in treatment individualization for ovarian cancer patients.

## Background

BRCA1 and BRCA2 are onco-suppressor genes involved in several crucial molecular events such as DNA repair, cell cycle regulation, apoptosis and genome integrity control [1]. More than 2,600 cancer predisposing mutations have been identified in BRCA1 and BRCA2 genes, on chromosome 17 and 13 respectively [2]. The genetic transmission follows a pattern of mendellian dominant inheritance with an approximate frequency of 1/800 in the Caucasians and 1/50 in the Ashkenazy jews. These mutations have been related to hereditary breast and ovarian cancer but also to prostate cancer, colon cancer, pancreatic cancer and male breast cancer [3]. Only 5-10% of all these cancers are actually related to one of several familial syndromes, the most common being the hereditary breast and ovarian cancer syndrome due to mutations of these tumor-suppressor genes [4]. Female carriers as compared to the general population have therefore an increased life-time risk to develop a breast and/or ovary cancer and are also at life-time risk of developing other tumors. Cancer predisposing mutations are considered to have a causative role in 65% of families with hereditary breast and ovary syndrome (HBOC syndrome) where are related to 60-80% of breast tumor cases and 20-40% of ovarian tumors [5]. In a 2003 report [6] the risk of breast and ovarian cancer for Ashkenazi women with inherited mutations in the tumor suppressor genes BRCA1 and BRCA2 has been estimated. On 1008 index cases, the lifetime risk of breast cancer among female mutation carriers was 82%. Moreover, in the recent years, the risk increased: breast cancer risk by age 50 among mutation carriers born before 1940 was 24%, but it was 67% among those born after 1940. In the same study, the lifetime risk of ovarian cancer was 54% for BRCA1 and 23% for BRCA2 mutation carriers.

A recent meta-analysis estimated the mean cumulative risk of developing breast and ovarian cancer by 70 years of age [7]. The mean breast cancer risk for BRCA1 and BRCA2 mutation carriers was 57% (95% CI, 47% - 66%) and 49% (95% CI, 40% - 57%) respectively [7]. The ovarian cancer risk for BRCA1 and BRCA2 mutation carriers was 40% (95% CI, 35% - 46%) and 18% (95% CI, 13% - 23%) respectively [7]. On these findings, it can be estimated that at least 15% of ovarian cancer cases are inherited tumors linked to a mendellian autosomic dominant inheritance of cancer predisposing mutations [8]. BRCA1/2 mutations account for more than 90% of hereditary ovarian cancer, whereas the remaining 10% is related to MLH1 and MSH2 mutations [9]. The identification of such genes in high risk female carriers provided valuable insights for the understanding of the natural history and pathogenesis of such diseases. It is an hard task to define the true prevalence of BRCA1/2 cancer predisposing mutations in the general population taking in account the variable presentation in different ethnic groups. In a recent study, it has been analyzed the prevalence of BRCA1/2 related to ethnicity in non-Ashkenazy women undergoing genetic testing from 1996 to 2006 [10]. Afro-american and latin-american women were diagnosed as carrier of BRCA1/2 mutations more commonly than women of european ancestry (15.6% *versus *12.1%) with a clear increase of BRCA1 mutations as related to ethnicity [10].

### BRCA1 and BRCA2 gene function and role in the DNA repair

Tumor cells lacking BRCA1 or BRCA2 function are highly genetically unstable. Important insights on BRCA1 functional role in the DNA repair mechanism is shown by physical interaction with RAD51 and BARD1 [11,12]. BRCA1 and BARD1 form a hetero-dimeric complex that functions in a variety of cellular processes, including transcriptional regulation, cell cycle progression and maintenance of X chromosome inactivation. Several findings suggest a specific role of BRCA1 and BARD1 in DNA repair [13]. Cell lines defective for BRCA1 or BARD1 exhibit genomic instability, are sensitive to DNA damaging agents and display defects in DNA double-strand breaks (DSBs) repair by homologous recombination (HR) [14]. Following exposure to DNA damaging agents, BRCA1 and BARD1 form a nuclear complex at sites of DNA damage where they colocalize with other DNA repair proteins such as RAD51 [15]. BRCA1 is also phosphorylated during the cell cycle and following treatment with genotoxic agents by the DNA damage checkpoint kinases ATM and ATR [16].

Both BRCA1 and BARD1 possess RING and BRCT domains. Recent studies suggest that the BRCT motifs may function as a phosphopeptide-binding domain that may be required for mediating protein-protein interactions with phospho-proteins and the N-terminal RING domains is responsible for tight association of the two proteins. This motif also confers E3-ubiquitin ligase activity raising the possibility that BRCA1/BARD1 hetero-dimer may specifically ubiquitinate proteins required for transcription, cell cycle and/or DNA repair [17].

On these findings, BRCA1 and BRCA2 appear to be functionally related to DNA repair mechanisms [18]. It is now clear that BRCA1 plays a critical role in the DNA damage recognition and in cell cycle checkpoints control that allows cell cycle progression only after DNA repair, avoiding genetic damage transmission in subsequent cell generations [19].

BRCA1 participates to a large multi-protein complex, the BRCA1-associated genome surveillance complex (BASC) [20], which acts as a sensor for DNA damage. BRCA2 has however a more direct role in DNA repair itself by driving RAD51 to the DSBs site [21]. Following recognition of DNA DSBs, BRCA1 is phosphorylated and leads to activation of the DSB repair by HR [22]. HR is an error-free pathway and operates the repair of DSBs in the late S and G2 phases of the cell cycle. An additional role of the HR is the repair of DSBs which occur for stalling of replication fork due to unrepaired single-strand breaks (SSB). In the absence of functional BRCA1 or BRCA2, cells become unable to undergo DNA repair by DSB and activate the non-homologous end joining (NHEJ) and single-strand non-homologous end-joining annealing (SSA), which are error-prone DNA repair pathways. Deficiency in DSB repair plays a crucial role in the chemo sensitivity profile of BRCA1- and BRCA2-deficient cells. It has to be considered that BRCA1 has also an important role in control of gene expression by the BRCT domain.

### Chemosensitivity of BRCA1/2-related ovarian tumors

#### 1. Preclinical findings

It is now well known that tumor cells lacking BRCA1/2 are highly sensitive to DNA damaging agents like platinum derivatives, as a consequence of impaired genomic damage repair which is induced by different mean [19,23]. Platinum compounds, through adduct formation at the DNA produce DBS, are specifically active in tumors with HR impairment for BRCA1/2 lack of function [24]. There is strong evidence from preclinical and clinical studies for a specific sensitivity of different BRCA1/2-related tumors to platinum derivatives. In 2003 our group has demonstrated a differential chemosensitivity profile *in vitro *of BRCA1-mutated HCC1937 breast cancer cells with bi-allelic loss as compared to the derivative clone HCC1937^WT^, where the BRCA1 expression has been reconstituted by transfection of a BRCA1 full length cDNA [25]. This study led to the conclusion that HCC1937 are highly sensitive to Cisplatinum (CDDP) as compared to Estrogen Receptor expressing and BRCA1-competent MCF-7 and BRCA1-reconstituted HCC1937^WT^. In the same work it was demonstrated that BRCA1-defective HCC1937 breast cancer cells were resistant to paclitaxel as compared to MCF-7 and HCC1937^WT ^cells. In a further study by our group a differential chemosensitivity of BRCA1-mutated HCC1937 human breast cancer cells to microtubule-interfering agents has been found [26] Quinn et al. in a similar cell system, where BRCA1 was reconstituted by a retroviral vector containing the full length cDNA and compared to parental cells infected with empty vector, demonstrated that BRCA1 functions as a differential modulator of chemotherapy-induced apoptosis. [27]. In a recent follow up study [28], our group evaluated the *in vivo *differential chemosensitivity of BRCA1-defective *versus *BRCA1-reconstrituted xenografts in SCID mice. In this mouse model, we confirmed a differential and higher activity of CDDP against HCC1937 BRCA1-defective xenografts. Furthermore, we demonstrated a major difference in the whole gene expression profile by cDNA microarray. Specifically, we found reduced expression of ERCC1 and RRM1 in HCC1937 *versus *BRCA1-reconstitued parental cells. Importantly, these two genes have been demonstrated to correlate, in previous studies in lung cancer, to an impaired response to CDDP treatment and to improved survival in patients undergoing treatment with CDDP/gemcitabine [29,30]. In addition, we found increased expression of mRNAs for RAD52 and XRCC1, genes related to DNA damage recognition. These latter findings strongly suggest a compensatory response to the impaired DNA damage repair in BRCA1 defective cells.

All together these experimental observations suggest that sensitivity to platinum derivatives inversely correlates to sensitivity to paclitaxel in BRCA1-defective breast tumor cells. Quinn et al. [31] have recently demonstrated a direct correlation between BRCA1 mRNA expression levels and overall survival in patients with ovarian cancer undergoing chemotherapy. These authors have shown that inhibition of BRCA1 expression in ovarian cancer cell lines increases cell sensitivity to platinum derivatives, while reduces the antitumor activity of taxanes. Subsequently they have evaluated BRCA1 mRNA expression in 70 tissue samples in sporadic ovarian tumors from patients which underwent treatment with platinum derivatives and they found that patients with low-intermediate levels of BRCA1 mRNA had a significantly better outcome in terms of overall survival (OS) as compared to high BRCA1 mRNA levels (57.2 months versus 18.2 months p = 0.0017). Finally high BRCA1 mRNA tumor carriers had a better survival if treated with taxanes even if statistical significance was not reached. It was concluded that BRCA1 mRNA expression levels correlate in sporadic ovarian cancer patients with OS and can be considered a predictive marker of treatment response. More recently the same authors have produced a systematic review [32] on all published studies on this topic from 1990 to 2008 leading to the hypothesis of a possible role of BRCA1 as biomarker predictive of treatment response in hereditary and sporadic ovarian tumors. The authors conclude that the identification of a functional deficit in BRCA1 and in related pathways is likely to provide information on treatment efficacy. Finally, the same authors have provided evidence that BRCA1 protein expression may be a predictive marker of chemotherapy response in sporadic epithelial ovarian cancer. They found that BRCA1 protein expression is associated with a better outcome of platinum/taxane combination as compared with CDDP alone, while when BRCA1 protein was not detected CDDP alone was as effective as combination chemotherapy. These data indicate again that CDDP alone may be highly effective in the case of BRCA1 impairment [33].

It is a common finding that human tumors highly sensitive to chemotherapy may become resistant [34]. Recent studies have shown that even the increased sensitivity to CDDP or Poly ADP Ribose Polymerase (PARP) inhibitors (see below) produced by BRCA1/2 gene mutation is not a stable trait [35]. Stacey Edwards et al have demonstrated that when pancreatic cells with BRCA2 inactivation become resistant to PARP inhibitors, novel BRCA2 isoforms were detected in the resistant line resulting from intragenic deletion of the c.6174delT mutation and restoration of the open reading-frame (ORF) [36]. Similarly Wataru Sakai et al. reported that secondary mutations in BRCA2 might reconstitute resistance to CDDP and PARP inhibitors in BRCA2 mutated tumors and that similar molecular mechanisms should be involved in clinical resistance to CDDP by ovarian tumors as demonstrated on clinical specimens [37].

All together these findings indicate that BRCA1/2 gene-mediated sensitivity to anticancer treatment can be reverted by escape mutations and that these important events must be taken in account for the design of novel therapeutic strategies in this specific setting.

#### 2. Clinical findings

Preliminary clinical evidence appears in line with preclinical *in vitro *findings and indicates that prospective clinical trials must be designed to clarify the clinical relevance of the differential sensitivity to anticancer drugs by BRCA1/2 mutated tumors.

In the last few years, the identification of individuals carriers of inactivating mutations on BRCA1 and 2 genes has been essentially directed to cancer prevention by the use of prophylactic surgery [38,39], preventive treatments or screening procedures different from general population [40-43]. Even with such measures, the onset of ovarian cancer in mutation carriers is a common event for failure of preventive strategies or because cancer predisposing mutations have been identified when the cancer has been already diagnosed. It is therefore often necessary the use of systemic chemotherapy regimens, which at present do not differ from those which are utilized in sporadic tumors. At present, ovary tumors are still a fatal disease in a high percentage of patients, due to late diagnosis for the lack of symptoms in early stage disease and partially for the intrinsic biologic aggressiveness. Systemic treatments are needed not only in advanced/metastatic disease but also in early cases and are based on the use of agents like platinum derivatives, taxanes, topotecan and liposomal doxorubicin.

The hereditary variants are characterized by typical clinical features, which often allow the selection of patients for genetic counseling and testing procedures, as early onset, multiple tumors especially in the breast, and family history for the same or other BRCA1/2 related tumors.

The increased risk of developing a ovary tumor in BRCA1/2 mutation carriers, assessed as 15,3% of the whole ovary cancer patients [44], has been related to the different functions of BRCA1 and 2 genes in the regulation of cell growth, genomic stability and repair of genomic damage by homologous recombination. Specifically, this last feature is the cause of the DSBs repair failure resulting in genomic instability and predisposition to neoplastic transformation for loss of function of BRCA1/2.

As regard to the clinical outcome of BRCA1/2-related breast tumors, several studies have been done in order to evaluate if germ-line cancer predisposing mutations might be useful for inclusion in different prognostic subgroups [43]. The majority of published studies has not produced proof of prognostic value of BRCA1/2 inheritance. Such studies present however several majors flaws for the retrospective design and because patients where not considered on the basis of stage, age, histology and residual disease after primary surgery[38].

A recent case-control study [45] included 779 jewish women affected by hereditary ovarian cancer who had undergone genetic testing for three Askhenazi founder mutations (BRCA1 185delAG, 5382insC; BRCA2 617delT). The design of the study was based on the comparison of mutation-positive *versus *mutation-negative ovarian cancer carriers in terms of long term outcome. The two groups were homogeneous for known prognostic, clinical and demographic factors.

This study clearly demonstrated a significantly better 5 years survival in mutation carriers as compared to non-carrier individuals (34.4% surviving in the non carriers *versus *46% in the BRCA mutation carriers p = 0.003). The survival gain occurred in advanced stages but not in early stages and at multivariate analysis, the prognostic weight of BRCA1/2 mutation was independent from age at diagnosis, histology and grading. Subgroup analysis demonstrated a better outcome for BRCA2-related *versus *BRCA1-related or BRCA-unrelated, while BRCA1-related did not behave favorably if compared to the two other subgroups. Interpretation of these subgroup analysis needs caution however at this point and confirmation in larger studies is eagerly awaited.

Data from this study appear of interest but it has to be considered that it is difficult to generalize these findings to non-Askhenazi population with a more heterogeneous mutational status, to evaluate the impact of BRCA1 *versus *BRCA2 as well as to speculate on the potential role of other confounding factors or modifier genes which might by themselves retain a prognostic weight. Moreover this study doesn't allow to understand if the survival advantage achieved in BRCA1/2 mutation carriers as compared to non carriers might be related to intrinsic biologic features or to a better response to treatment. In any case this landmark study provides proof of principle that ovarian cancer arising in BRCA1/2 mutation carriers is a different disease.

Norah Kauff [38,46] has discussed these important findings in a provocative *Journal of Clinical Oncology *editorial. The identification of BRCA1/2 related ovarian cancer as a distinct disease has important implications. Imbalance of BRCA1/2 related ovarian tumors in the arms of a randomized trial will introduce a powerful bias. It can be therefore inferred that all ovarian cancer patients enrolled in prospective randomized trials might be stratified on the basis of presence or absence of BRCA1/2 cancer predisposing mutations. These points merit further discussion. BRCA1/2 testing is an expensive procedure and has important ethical/consent implications. We think that prospective genetic testing cannot be performed in unselected individuals. We think however that enrollment in ovarian cancer clinical trials should be reserved to clinical centers offering genetic counseling to all ovarian cancer patients. Genetic testing based on pretesting counseling will allow the identification of most BRCA1/2 related tumors. In any way, Kauff's point needs to be considered in the planning of future clinical research in ovarian cancer.

### BRCA-ness in the current scenario of management of ovarian cancer

Important information has been derived from a mono-institutional case-control study recently reported by Tan et al. [46]. The authors confirm a more favorable outcome in BRCA1/2 mutation carriers with a significant advantage in OS and demonstrate a differential chemosensitivity. A clear advantage in the treatment free interval (TFI) is achieved in BRCA1/2 related tumors when patients are treated with platinum-containing regimens in different lines of treatment (median 15 months for BRCA1/2 versus 9 months for non BRCA1/2, first to second line p = 0.001; median 15 months for BRCA1/2 versus 5.6 months for non BRCA1/2 p = 0.002 second to third line). The better TFI is paralleled by an higher level of radiological responses (Overall Response Rate, ORR 95.5% for BRCA1/2 mutated *versus *59.1% for non BRCA1/2 p = 0.002 in first line treatment). On the other hand, BRCA1/2 tumors did not show an increased benefit from non platinum-based chemotherapy regimens (median TFI 4 months for BRCA1/2 versus 6 for non BRCA1/2 second to third line p = 0.831). This study indicates that BRCA1/2 related ovarian cancers have a better outcome because are intrinsically highly sensitive to platinum containing chemotherapy. The authors provide evidence for a "BRCA-ness" syndrome in BRCA1/2 mutation carriers which includes serous histology, high response to first and subsequent lines of platinum-based treatment, longer TFIs between relapses, and improved OS.

### BRCA-ness in the evolving scenario

**Figure 1:**
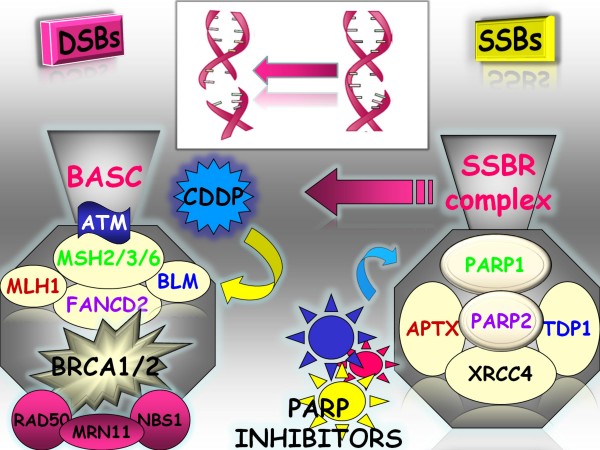
**DNA repair defects and therapeutic intervention in BRCA1/2 defective tumors**. Following DNA damage poly(ADP-ribose) polymerases (PARP), specifically PARP-1 and PARP-2, are activated and bind to the exposed Single Strand Breaks (SSBs). Pharmacological inhibition of PARP1 with PARP-inhibitors leads to a block in the repair of SSBs, resulting in the blockage of replication fork and subsequent conversion of damage in DSBs. In hereditary cancers harboring BRCA1/BRCA2 mutations, this system is inefficient and therefore the tumor cells lacking this survival mechanism undergo cell death. The antitumor activity of PARP inhibitors may be enhanced by combination with chemotherapeutic agents which induce direct damage to DNA, such as platinum derivatives.

The pharmacologic interference with alternative genomic damage repair pathways as those related to single strand break repair (SSBRs) might be of relevance for hereditary BRCA1/2 related tumors [47-49]. It is a recent finding the identification of an enzyme family the PARPs, which includes different molecules with different activity and function, some of them strictly related to the Base Excision Repair (BER), which is involved in the SSBRs. PARP1 is the most studied enzyme in this family and is involved through BER activation in the cellular response to genomic damage produced by geno-toxic stress. PARP1 binds to the sites of damage at the single strand and catalyzes the synthesis and subsequent transfer of chains of poly-(ADP)-ribose (PAR) to carboxylic groups of several proteins, including PARP1 itself. This enzyme uses nicotinamide adenine dinucleotide (NAD^+^) as substrate to synthesize poly(ADP)-ribose and leads to a series of linear or branched polymers of PAR [21]. PARP1 contains three functionally distinct domains: an amino-terminal DNA-binding domain (DBD), an auto-modification domain (AD), which is linked to BRCT-domain of BRCA1, and a carboxyl-terminal PARP homology domain, that includes the catalytic domain (CAT) responsible for PAR formation [50]. The ADP-ribosilation creates target sites of SSBs negatively charged that recruit the enzymes needed to form BER multiprotein complex, such as: XRCC1(X-ray repair cross-complementing 1), DNA ligase III and DNA-polymerase. Following poly-(ADP)-ribosylation, PARP1 loses affinity for DNA, detaches and exposes sites of damage, thereby allowing access to DNA to repair enzymes; PARP1 subsequently undergoes degradation. It has been hypothesized that, in addition to SSB-repair PARP1 plays also a critical role in double-strand breaks(DSBs)-repair, although at present no direct functional correlation has been demonstrated with the nonhomologous end joining (NHEJ) or HR. PARPs system is involved not only in repair mechanisms, but also in transcriptional regulation, plays a key role in regulation of cell death and survival and represents an important regulatory factor in the molecular events leading to the development of cancer or inflammatory disease. *In vitro *and *in vivo *studies support the use of PARP inhibitors not only as chemo and radiosensitizing agents, but also as selective agents in those tumors carrying specific functional defects in DNA repair mechanisms, such as cancers harboring specific mutations of BRCA1 and 2. Indeed, when SSB repair is inactivated by PARP1 pharmacological inhibition during S-phase, DNA DSBs are induced. This latter effect may confer synthetic lethality to cells with defective homology-directed DSB repair like cells with BRCA1 and BRCA2 deficiency (Figure 1) [21,51,52].

The finding that BRCA1/2 deficient cells are highly sensitive to PARP inhibition [53] has opened a new avenue of research for treatment and prevention of tumors arising in the context of BRCA1/2 mutation or which might have somatic impairment of such pathways, such as basal-like breast cancer. Rottemberg et al. have recently demonstrated in a genetically engineered mouse model of BRCA1 related-breast tumor that the PARP inhibitor AZD2281 (olaparib) is highly effective alone or in combination to CDDP [54]. Several PARP inhibitors are presently available and under investigation in clinical trials (Table 1). A seminal phase I trial has provided evidence that olaparib is well tolerated, inhibits PARP activity in surrogate samples and also in tumor samples and exerts activity against BRCA1/2 related cancer [55]. Very recently a phase II trial with olaparib has been reported in BRCA1/2 deficient ovarian cancer. Olaparib has been given orally in 28 days cycles initially at the MTD 400 mg bis in die (bd) (33 patients) and subsequently at 100 mg bd (24 patients) The confirmed RECIST ORR was 33% at 400 mg bd and 12.5% at 100 mg bd. These data clearly show that Olaparib is highly effective in advanced pretreated BRCA1/2 related ovarian cancer [56]. Olaparib appears therefore an attractive option for use in earlier phases of disease and to be evaluated in combination with platinum derivatives on the bases of important preclinical studies. Results from ongoing trials are eagerly awaited.

**Table 1:** Parp-inhibitors on Clinical Trials

**Parp-inhibitors**	**Pharmaceutical Company**	**Clinical development**
Olaparib (AZD2281)	AstraZeneca	Breast, ovarian and prostate cancer BRCA1-BRCA2 related Breast cancer Ovarian cancer Pacreatic cancer Colorectal cancer Melanoma neoplasm Unspecified adult solid tumors

BSI-201	BiPar Sciences Inc.	Uterine cancer Brain neoplasm Triple negative breast cancer

ABT-888	Abbott	Metastatic Melanoma Skin cancer Breast Cancer Ovarian Cancer Primary Peritoneal Cancer Fallopian Tube Cancer

MK 4827	Merck & Co. Inc.	BRCA-related ovarian cancer Ovarian cancer Solid tumors

## Conclusion

All together these findings introduce a provocative novel scenario where BRCA1/2 carcinogenetic process in the hereditary setting produces novel opportunities for pharmacological intervention. Apart novel drugs like PARP inhibitors, these findings may allow a different and more rational approach for the treatment of BRCA1/2 related ovarian tumors by currently available drugs. The study by Tan et al[46] clearly demonstrates that CDDP resistance in BRCA1/2-related tumors is a late event and patients experience a long treatment free interval after CDDP-based treatment. The common finding that paclitaxel appear less effective in preclinical models of BRCA1/2 models would suggest a more rational first line treatment with CDDP/gemcitabine combination or even with carboplatin escalated doses in order to achieve the maximal benefit in advance of the occurrence of escape mutations like those recently described in BRCA2 gene. All these approaches need of course to be explored in well designed prospective clinical trials. The finding by Quinn et al[31] and by Carser et al. [33] that low BRCA1 mRNA and protein expression is predictive of specific benefit of platinum based chemotherapy, while high BRCA1 mRNA might predict for benefit of taxane treatment, might allow to explore the potential advantage of molecular marker-based treatment assignment compared to conventional assignment. This topic is prospectively evaluated in non small cell lung cancer (NSCLC) by Rosell and coworkers[29,30,57].

Treatment tailoring of ovarian cancer on the genetic background appears now to be based on a robust rationale from preclinical and clinical evidence and it is time to undergo evaluation in well designed prospective trials.

## Competing interests

The authors declare that they have no competing interests.

## Authors' contributions

PST, MV and PFT participated in drafting the full manuscript and creating figures. FB, IC, MA and MTDM participated in substantial contribution to conception and revising it critically for important intellectual content. All authors read and approved the final manuscript.
